# Oncogenic Role of miR-217 During Clear Cell Renal Carcinoma Progression

**DOI:** 10.3389/fonc.2022.934711

**Published:** 2022-07-22

**Authors:** Jose María Zamora-Fuentes, Enrique Hernández-Lemus, Jesús Espinal-Enríquez

**Affiliations:** ^1^ Computational Genomics Division, National Institute of Genomic Medicine, Mexico City, Mexico; ^2^ Centro de Ciencias de la Complejidad, Universidad Nacional Autόnoma de México, Mexico City, Mexico

**Keywords:** clear cell renal carcinoma, gene co-expression networks, WKN2, GALNTL6, IGF2BP2, miR-217, cancer progression stages, renal carcinoma progression

## Abstract

Clear cell renal carcinoma (ccRC) comprises a set of heterogeneous, fast-progressing pathologies with poor prognosis. Analyzing ccRC progression in terms of modifications at the molecular level may provide us with a broader understanding of the disease, paving the way for improved diagnostics and therapeutics. The role of micro-RNAs (miRs) in cancer by targeting both oncogenes and tumor suppressor genes is widely known. Despite this knowledge, the role of specific miRs and their targets in the progression of ccRC is still unknown. To evaluate the action of miRs and their target genes during ccRC progression, here we implemented a three-step method for constructing miR–gene co-expression networks for each progression stage of ccRC as well as for adjacent-normal renal tissue (NT). In the first step, we inferred all miR–gene co-expression interactions for each progression stage of ccRC and for NT. Afterwards, we filtered the whole miR–gene networks by differential gene and miR expression between successive stages: stage I with non-tumor, stage II with stage I, and so on. Finally, all miR–gene interactions whose relationships were inversely proportional (overexpressed miR and underexpressed genes and *vice versa*) were kept and removed otherwise. We found that miR-217 is differentially expressed in all contrasts; however, its targets were different depending on the ccRC stage. Furthermore, the target genes of miR-217 have a known role in cancer progression—for instance, in stage II network, GALNTL6 is overexpressed, and it is related to cell signaling, survival, and proliferation. In the stage III network, WNK2, a widely known tumor suppressor, is underexpressed. For the stage IV network, IGF2BP2, a post-transcriptional regulator of MYC and PTEN, is overexpressed. This data-driven network approach has allowed us to discover miRs that have different targets through ccRC progression, thus providing a method for searching possible stage-dependent therapeutic targets in this and other types of cancer.

## Introduction

The global incidence of renal cell carcinomas (RCC) has notoriously increased since 2008, exerting an important burden in both individuals and health systems (1). A number of basic and clinical endeavors have been implemented to try to alleviate this situation. Important efforts have been made in searching for key regulators in the development of this disease. Oncogenes and tumor suppressors such as VHL (3p26), FH (1q42.1), MET (7q34), or FLCN (17p11.2) genes have been studied in different types of RCC. These have been associated with different syndromes and inheritance patterns (2).

However, up to 70% of RCC cases correspond to the clear cell subtype (ccRC). The progression in this tumor subtype is commonly initiated by mutations in VHL. Some transcription factors are accumulated due to VHL inactivation, which induces the expression of vascular endothelial growth factor (VEGF). Therefore, ccRCCs are often highly vascularized and respondent to anti-angiogenic therapy (3). Subsequent mutations commonly arise in BAP1/PBRM1/SET2/KDM5C, giving rise to DNA repair defects. These genes are then considered as gene drivers for ccRC evolution. Moreover, activation of the PI3K pathway promotes metastases (4). Considering that up to a third of cases will present metastases, the importance of determining with a higher accuracy the molecular factors underlying the progression of ccRC is undeniable. Additionally, there is evidence that VHL inactivation in humans and mice does not directly induce ccRC tumorigenesis (5).

Regarding immune responses, the relationship of the microenvironment with ccRC progression is not clear. However, efforts have been made to find the patterns of macrophages and T cells, characterized by a wide diversity, both in phenotypes and responses (6). To overcome this challenge, efforts have been made to find markers of the different elements of immune response—for instance, in (7), it was found that individuals with inflammatory responses enriched for BAP1 have a worse prognosis. PBRM1 has also been found in both animal models and in human samples with decreased immune infiltration. It has also been observed that PBRM1-knocked-out tumors were more resistant to anti-PD-1 antibody (8).

Tumor microenvironment heterogeneity is indeed just partially responsible for the complexity behind ccRC responses. Regulatory elements and epigenomic modulators are known to be also playing relevant roles—for instance, it has been argued that micro-RNAs (miRs) appear to regulate more than 60% of human genes (9). Furthermore, aberrant expression patterns of miRNA have been reported in many human cancers (10). A number of these genes are considered as key factors in cancer development pathways (11). To mention just a few, miRs such as miR-646, miR-21, and miR-204 have been implicated in the development and progression of renal cell carcinoma (12). miR families, such as miR-200 family, have also been reported to be strongly dysregulated in metastases and met-like primary tumors (13, 14).

The effects of miRNAs over gene regulation are complex and highly context dependent, varying by cell type as well as by the severity and persistence of conditions in cell signaling and other processes, including genomic damage (15). The effects that miRs exert on gene expression are often mostly attributed to the miR–mRNA 3′ untranslated region (UTR) interactions. These interactions lead to target post-translational inhibition or degradation. However, focusing on this mechanism to design miR therapeutics is likely proven to be too simplistic, owing in part to other emerging micro-RNA mechanisms, which include decoy activity and 5′ UTR and direct DNA regulatory activities.

Alternatively, miRNAs can be associated to the development of tumor-suppressive and oncogenic functions, and their ability to modulate different genes may be also context dependent (3). Specifically, miR–gene regulation may repress or promote transcription (non-canonical) or translation (canonical) (15).

It is widely known that a single miR can regulate several mRNAs and that a single mRNA transcript can be targeted by several miRs (16). To broadly understand the intrinsic complexity of a miR–gene interaction, developing integral approaches that combine different sources of information becomes mandatory. Additionally, it is necessary to take into account the complex nature of miR–gene regulation and its many associated mechanisms.

Abnormalities in cell behavior that involve the dysregulation of gene and miR expression have been argued to play relevant roles in triggering carcinogenic processes. Several studies have confirmed, for instance, that overexpression of miRs has the potential to promote cancer development [for a broad review, see (17–19)]—for example, miR-203 has been related to follicular grow factor 2 (FGF2) and CAV1 as a downstream regulator, affecting pathways such as PI3K/AKT/mTOR (20). There is also evidence that miRs such as miR-141, miR-200a, or miR-200b may serve as drivers in the epithelium-to-mesenchymal transition and the complementary process, the mesenchymal-to-epithelium transition, by inhibiting the expression of VIM, ZEB1, or ZEB2 genes (14). Processes such as proliferation, migration, invasion, or apoptosis can be altered by miR-203 by targeting FGF2 (12).

Despite the ever-growing evidence of the role that miRs exert on the oncogenic process, a comprehensive list of oncomiRs or tumor suppressor miRs, particularly for ccRC, is still lacking (11).

Building such a comprehensive catalog is indeed easier said than done. Important steps have been taken, however, in this direction. With the advent of next-generation sequencing, gene expression profiles have been extensively used to discover crucial features based on the expression of certain genes that may drive the reconfiguration of the transcriptional program, often leading to dramatic effects on the phenotype (21–25).

Although the importance of genetic expression in cancer is out of discussion, it is also clear that the gene regulation during the carcinogenic process is strongly altered by several components. Additionally, the gene expression landscape often does not provide information on how those genes are regulated (26).

To overcome the latter challenge, a common approach used for high-throughout-derived datasets is the gene co-expression network (GCN). These networks are commonly inferred by correlating the expression profile of gene couples with multiple samples. GCNs offer a framework that allows the analysis of global changes in a given phenotype, such as cancer. With this approach, the statistical dependency of a given gene can be quantified by the expression of any other gene (27–31).

In relation to ccRC, we recently demonstrated that differential gene expression profiles are quite similar between progression stages; however, the gene co-expression networks observed in those stages resulted different in terms of structure and also the associated biological processes involved in such networks (32).

The evidence of the role of miRs in the rise and development of cancer, in particular for clear cell renal carcinoma, is increasing. miR alterations may be key factors in the development and progression of ccRC to more advanced stages. However, the specific role of miRs during the progression stages of ccRC is still unknown.

In order to evaluate the role of the miRs–gene relationships in ccRC progression, here we implemented a three-step method for constructing miR–gene co-expression networks for the four progression stages of clear cell renal carcinoma as well as for healthy renal tissue, with data obtained from The Cancer Genome Atlas (TCGA)-GDC consortium.

In the first step, we inferred all miR–gene co-expression interactions of each progression stage of ccRC and for the healthy renal tissue. In the second step, we filtered the whole miR–gene networks by differentially expressed miRs and genes. We assessed the differentially expressed genes between non-tumor adjacent-to-tissue control samples and each progression stage. However, to establish the progression between stages with higher accuracy, we calculated the differentially expressed miRs and genes from contiguous stages: stage I *vs*. control, stage II *vs*. stage I, and so on.

Once each network was constructed, we conserved the miR–gene interaction whose relationships were inversely proportional (overexpressed miR and underexpressed genes and *vice versa*); otherwise, we removed them. Finally, we observed the shared genes and interactions between cancer stages and also those genes and interactions that resulted unique for each stage. With this data-driven network sifting, we were able to discover miRs that have different targets through the clear cell renal carcinoma progression, thus providing a method for searching possible therapeutic targets in ccRC and other types of cancer.

## Materials and Methods

**Figure 1:**
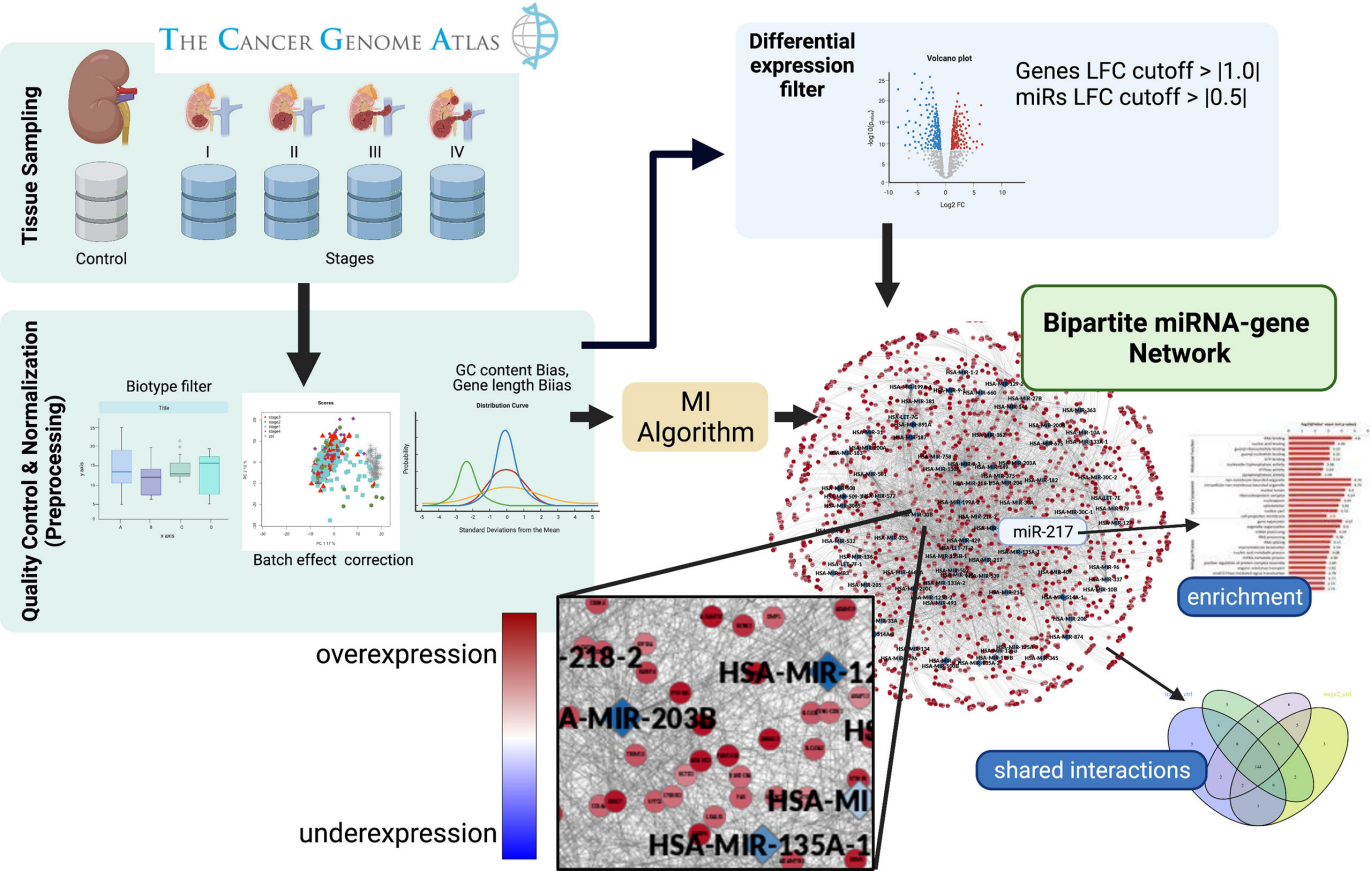
Graphical pipeline. Firstly, sequencing data was obtained from the TCGA-GDC consortium. Secondly, a pre-processing phase was performed, where raw counts were filtered and normalized. Differential expression was calculated to retrieve genes and miRs whose expression became altered between contiguous stages. A bipartite co-expression network (miR–gene) for each progression stage was inferred. After that, we conserved those miR–gene interactions with miRs and genes with opposite differential expression. Finally, an enrichment analysis of the relevant genes obtained by the aforementioned pipeline was performed.

In order to carry out the research program just outlined, we have implemented a streamlined analytics methodology. A graphical representation of the workflow followed is shown in **Figure 1**. In the following subsections, we will expound on the different aspects of the workflow just presented.

### Data Acquisition

ccRC RNA sequencing data was obtained from TCGA collaboration (33–35). To obtain the gene expression profiles for each progression stage, we started by downloading RNA-Seq level 3 gene expression files for ccRC samples. Additionally, we downloaded the miR profiles for the same samples; therefore, all samples were paired RNA/micro-RNA, and the corresponding metadata is indeed harmonized. Hence, we compiled two main datasets (1): miR expression quantification (reads per million) and (2) isoform expression quantification, which contains detailed information about the transcribed species (as coordinates mapped) for each transcript. This can be used to get mature micro-RNA information.

The indexes of both datasets were harmonized to match patient codes as a master key for agglomerating RNAseq and micro-RNA raw counts. A summary of pre-processed data can be seen in **Table 1**.

**Table 1:** Number of harmonized cases for each stage of ccRC and NT.

	NT	st_I_	st_II_	st_III_	st_IV_
Number of samples	71	251	55	122	81

### Clinical Information

We processed the clinical information directly from the TCGA-KIRC project. We categorized all samples by its *tumor_stage* variable. Samples with not-reported stages were removed. The TCGAbiolinks library (V 2.24.1) was used to retrieve data from TCGA.

### Pre-processing

We pre-processed gene and miR data as follows: (1) we removed genes without annotation in the BioMart Database, (2) we removed genes with more than 50% of zero counts per sample, and (3) genes with a mean expression of less than 10 counts were also removed. For bias correction, we used the EDASeq R-package (V 2.30.0) (36). In brief, we removed biases in GC content, gene length, and biotype. Finally, in order to correct for possible batch effects, we used the ARSyN method, implemented in R as a function of NOIseq library (V 2.40.0) (37).

After all filters were implemented and the bias removal was performed, the total number of miRs for analysis was 275; meanwhile, the total of genes was 16,224. Those were the entities used to infer miR–gene networks and to perform differential expression analyses. A summary of phenotypes, units of counts, and size of genes and miRs is portrayed in **Table 2**.

**Table 2:** Summary of genes and miRs.

	Genes	miRNAs
Size	16,224	275
Units	HTSeq - counts	Reads-per-million-miRNA-mapped
Phenotypes	5	5

### Differential miR and Gene Expression

Differential expression analysis was implemented by using the DESeq R package (V 1.8.3) (38). Here we considered differentially expressed genes (DEGs) with the following filters: |LogF C| > 1.0 and FDR-corrected d p − value < 1e − 5 . Meanwhile for differentially expressed miRs (DEMs), the filters were |LogF C| > 0.5 and p−value < 1e−5. It is worth noticing that the logFC cutoffs depend on the empirical data distributions and the associated dynamic ranges of the measurements of the variables. Even though both RNASeq and miRNASeq were performed with roughly the same technology (Illumina NGS Sequencing), there are indeed differences in the capture rates, the variant calls and annotations, and other issues of the experimental methodologies. Even more important, there are differences in the natural abundance of these two types of transcripts in the samples.

We compared the non-tumor (NT) dataset with all progression stages (*st_I_ , st_II_ , st_III_ , and st_IV_*). Additionally, in order to track down the evolution of the tumor progression, we also performed differential expression analysis between contiguous stages (progression contrast) NT- *st_I_, st_I_ -st_II_*, and so on. To visualize the DEGs and DEMs, we constructed volcano plots for each contrast with the default specifications of EnhancedVolcano (V1.14.0) package (https://github.com/kevinblighe/EnhancedVolcano).

We observed the number of DEGs and DEMs which appeared for each contrast. We also calculated those unique DEGs and DEMs for each contrast as well as those shared DEGs/DEMs in all contrasts. The code to develop these analyses can be found at the following repository: https://github.com/josemaz/kirc-mirna.

### Network Inference

To analyze the potential role played by miRs in the gene expression program, we inferred five miR–gene networks, one for tumor-adjacent-healthy-tissue (NT) samples, and one for each tumor progression stage. All networks were inferred by using mutual information (MI) as a statistical dependence measure. MI was calculated over the expression values for all miR–gene couples (275 × 16, 227 ≈ 4.5 × 10^6^ pairwise interactions) for each phenotype. We implemented a multi-thread miR–gene co-expression calculation based on the ARACNe algorithm (27). The code to infer such MI-based networks can be found at https://github.com/josemaz/aracne-multicore.

### Network Filtering and Visualization

In order to find dysregulated genes targeted by micro-RNAs, we used both DEGs and DEMs as network filters. Briefly, we conserved the 100,000 highest miR–gene MI interactions to capture the most relevant co-expression relations for any given phenotype. We conserved only those miR–gene interactions in which the micro-RNA and its target have opposite differential expressions: overexpressed miR and underexpressed gene and *vice versa* looking for canonical miR–gene interactions.

Finally, we analyzed the fraction of conserved miR–gene interactions and the fraction of unique interactions for each phenotype. Network visualizations were performed with Cytoscape 3.8.2 (39).

## Results and Discussion

### NT and ccRC Contrasts

We performed a multi-group comparison between control and each progression stage. We found a larger number of over-expressed genes and miRs than that of underexpressed ones. **Table 3** shows the comparison between miRs and differentially expressed genes between non-tumor (NT) and each progression stage of ccRC (*st_I_, st_II_, st_III_, and st_IV_*). Interestingly, the number of DEGs increases with progression stages; this may suggest that the whole gene regulatory program becomes more disrupted as the tumor evolves to later stages.

**Table 3:** DEGs and DEMs for each progression stage as compared with non-tumor adjacent tissue-derived samples.

	NT–st_I_	NT–st_II_	NT–st_III_	NT–st_IV_
Underexpressed genes	1,946	2,012	2,106	2,187
Overexpressed genes	2,187	2,238	2,587	2,630
Overexpressed miRs	88	87	96	91
Underexpressed miRs	87	87	88	88
**All genes**	4,133	4,250	4,693	4,817
**All miRs**	175	174	184	179

The large difference of DEGs and DEMs between NT and *st_I_*, compared with the rest of contrasts, may be due to the recruitment and accumulation of several different cancer-associated cell types, aside from the intrinsic genomic alterations of cancer cells with respect to normal ones.

Despite the large number of DEGs and DEMs, unique differentially expressed genes or miRs are quite scarce. **Table 4** shows the number of unique DEGs and DEMs per contrast. As observed, the number of unique DEGs/DEMs per contrast is almost 40 times lower than the total amount of DEGs/DEMs.

**Table 4:** Unique differentially expressed genes and miRs for each non-tumor stage contrast.

	NT–st_I_	NT–st_II_	NT–st_III_	NT–st_IV_
Overexpressed genes	52	54	61	189
Underexpressed genes	56	58	30	141
Overexpressed miRs	2	2	4	4
Underexpressed miRs	1	1	1	3

These results become of particular interest because it appears that most of the DEGs/DEMs are conserved throughout the whole evolution of the disease. However, as we have previously observed in gene–gene co-expression networks (32), differential expression is not sufficient to explain the evolution of the early stages to the more advanced ones.

### DEGs and DEMs Are More Abundant Between Control and Stage I Than in Any Other Contrast

Although the differential expression between control and progression stages provides information regarding those genes and miRs that may exert influence on the acquisition of oncogenic traits, a comparison between contiguous stages can be, in some sense, more revealing since it represents the evolution of the gene expression program along tumor progression.

**Figure 2:**
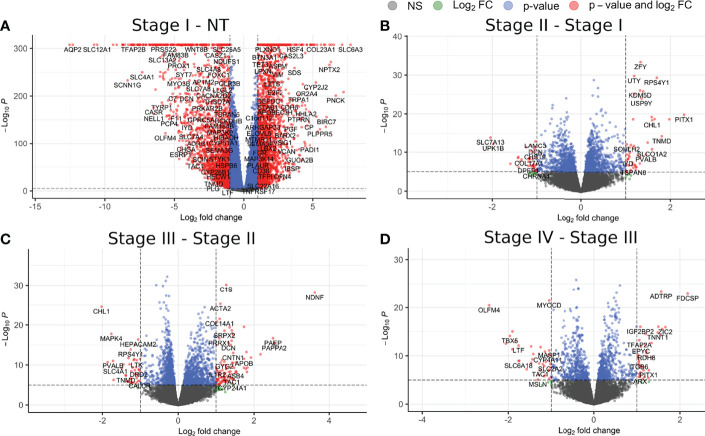
Differentially expressed genes for each contiguous stage of ccRC. **(A)** Contrast between control and stage I; **(B)** stage I V, stage II; **(C)** stage II V, stage III; **(D)** stage III V, stage IV. Red circles represent genes with a |log_2_FC| >1 and a *p*-value **<**1e-5; circles depicted in green take account for those genes with a |log_2_FC| >1 but *p*-value **<**1e-5; those genes with a |log_2_FC| <1 but a *p*-value **<**1e-5 are depicted in blue. Finally, those genes with values lower than those thresholds are depicted in gray. It becomes evident that the contrast with more DEGs is the one between NT and st_I_.

**Figure 3:**
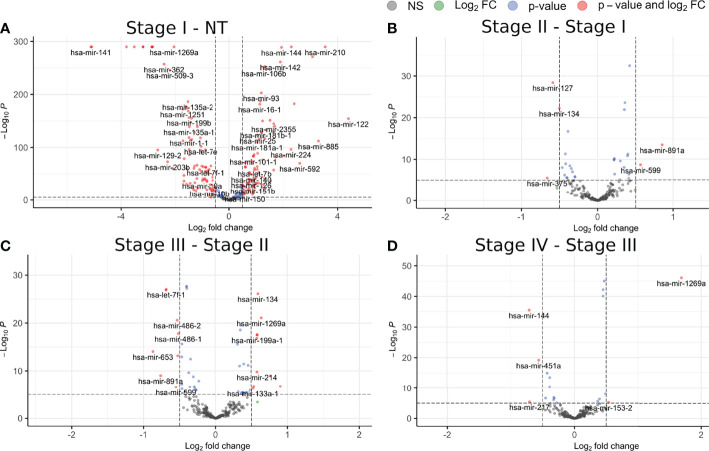
Differentially expressed miRs for each contiguous stage of ccRC. **(A)** Contrast between control and stage I; **(B)** stage I V, stage II; **(C)** stage II V, stage III; **(D)** stage III V, stage IV. The color code is the same as that in **Figure 2**.

To further investigate on this, we performed a differential expression analysis between sequentially contiguous stages. In **Figures 2**, **3**, we can observe volcano plots showing the DEGs and DEMs between the consecutive stages of ccRC evolution: NT − *st_I_ , st_I_ − st_II_ , st_II_ − st_III_*, and *st_III_ − st_IV_*. **Supplementary Material S1** shows the shared and unique genes/miRs for each contrast.

**Figure 2** shows the volcano plots for the DEGs. As can be seen, the contrast with the larger number of DEGs is that between NT and *st_I_* , with a total of 2,187 overexpressed genes and 1,946 underexpressed ones. The following contrasts had a number of DEGs more than 100 times lower than the first one. Analogously, in **Figure 3**, we can observe a similar behavior for DEMs. According to these results, in ccRC, the main changes in the gene and miR regulatory programs occur in the initial phase of tumor evolution.

In the latter contrasts, both DEGs and DEMs show specific differences in their expression—for instance, mir-155 (considered as miR regulator of VHL) (40, 41) is overexpressed in the T-stage1 comparison: However, in the following contrasts it is not differentially expressed.

To notice, in the latter contrasts, for both cases of miRs and genes, the list of DEGs and DEMs is different for each contrast. In **Supplementary Material S2**, we provide all contrasts between NT and all ccRC progression stages as well as between sequentially contiguous stages.

The resulting overexpressed and underexpressed miRs and genes were then used to construct the miR–gene networks for each phenotype (NT and the four stages). We conserved only those miR–gene interactions between DEGs and DEMs with an opposite differential expression trend (potentially corresponding to the canonical mechanisms of miR–gene regulation).

### miR–Gene Networks Are Mostly Stage Specific

**Figure 4:**
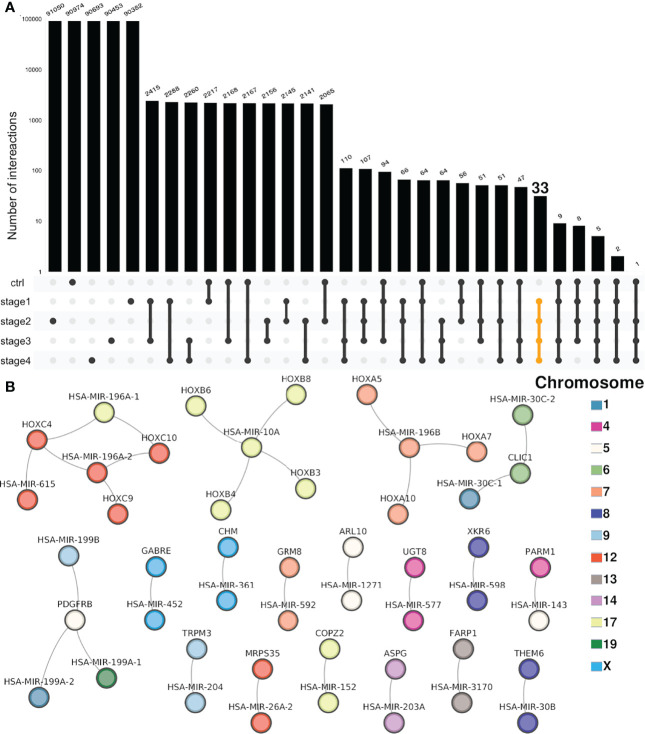
Intersection of miR–gene co-expression networks. **(A)** Each bar in the UpSet plot represents the number of interactions in the selected set, represented by linked points below the bars (log scale). Above each bar, the number of interactions is shown. The first five bars account for unique interactions. From the sixth bar onward, each one of them shows the number of shared interactions between two or more networks. At the right side, the set of shared interactions between the four CCRC progression stages (but not NT) is highlighted in yellow. **(B)** The 33 shared interactions between the four progression stages but not shared with the non-tumor network are depicted. In the figure, the color of the nodes represents the chromosome where miRs and genes are located.

**Figure 4** shows an upset plot of the shared miR–gene interactions for each stage. In stark contrast with the high number of shared genes among DEGs and DEMS, in the case of miR–gene networks, there is only a small subset of interactions that are shared between networks. More than 90% of the miR–gene interactions are unique for each network.

This result was seemingly counter-intuitive at first since the number of shared genes and miRs between contrasts was very high. However, the miR–gene regulatory programs, as represented by high-confidence co-expression networks, are indeed highly specific for each progression stage.

A concomitant result derived from the uniqueness of miR–gene interactions for each progression stage is that there is a small set of shared interactions between cancer stages but not shared with the NT network. Only 33 interactions are common for the four stages and not presented in the non-tumor phenotype.

By looking at those interactions, it can be appreciated that practically all of them correspond to miRs and genes that belong to the same chromosome. Furthermore, they belong to the same cytoband (**Supplementary Material S6**).

Interestingly, among the most connected miRs corresponding to the miR-196 family are the following: miR-10A and miR-196A-1 (Chr17q21.32), miR-196A-2 (Chr12q13.13), and miR-196B (Chr7p15.2). As can be observed in **Figure 4B**, those miRs (upper part of the network) are associated with HOX genes which belong to the same location than the said miRs: HOXC9, HOXC10, and HOXC11 are located at Chr12q13.13. Analog is the case of HOXA5, HOXA7, and HOXA10 (Chr7p15.2) or HOXB3, HOXB4, HOXB6, and HOXB8 (17q21.32).

The role of HOX genes in the rise and development of several types of cancer has been extensively reported (42–44). Additionally, the role of the miR-196 family has been also described in different carcinomas (45–47). The fact that the miR-196-HOX genes complexes are shared between all progression stages but absent in the non-tumor network, may indicate the role of these relationships in ccRC progression.

It is worth to noticing, genes shared by non-tumor (control) and stages were: HOXA9, MEST, TENM4, ARPP21, DIO3. As it was mentioned, HOAX genes have an important role in cancer. The loss of imprinting of MEST gene has been linked to certain types of cancer and may be due to promotor switching. However, all those genes play a critical role in mammalian development as a common feature.

Finally, the neighboring location of miRs and genes observed in **Figure 4B** has been previously described in gene–gene co-expression networks for breast cancer (28, 30, 48), lung cancer (31), and also in clear cell renal carcinoma progression stages (32). In this case, where the inferred networks are obtained by correlating the micro-RNA expression with the gene expression, the effect of loss of long-distance co-expression is not appreciated in the whole networks. However, in the 33 (out of 100,000 for each stage) cancer-shared interactions, we observe not only miR–gene interactions with molecules from the same chromosome but also the same cytoband and, furthermore, contiguous locations in terms of start positions (**Supplementary Material S6**).

After observing the location of miRs and genes in the shared network, we argue that the appearance of intra-cytoband interactions in cancer-exclusive phenotypes could be related to an anomalous transcriptional event which allows to have similar expression patterns between microRNAs and gene transcripts. Experimental corroboration, however, is needed to fully elucidate the role of those interactions. Additionally, the HOX–miR-196 complex should also be investigated in order to provide a possible explanation of those development-related genes in ccRC progression.

### miR–Gene Networks Are Different Between Stages, Both in Size and Composition

As previously stated, we inferred five networks (one for each phenotype), one network for NT, and one network for each ccRC progression stage. To construct all networks, we calculated the mutual information measure between miRs and genes by using the expression matrices for miRs and genes in all stages (see **Table 1**).

**Figure 5:**
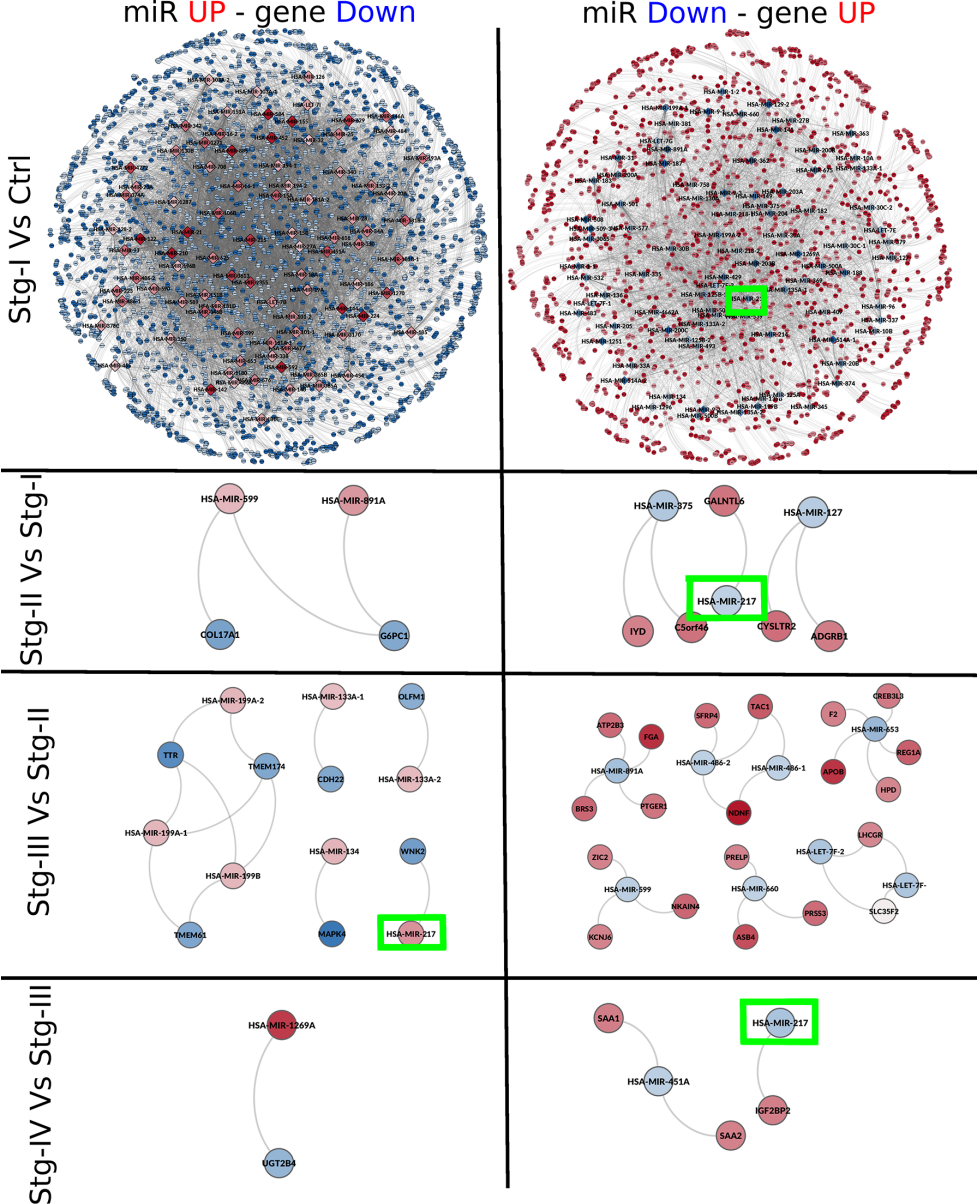
miR–gene networks for each progression stage. In this figure, we can observe networks inferred by mutual information between the expression of miRs and genes for each progression stage. Networks are placed from top to bottom according to the progression stage. The contrast used to depict each network is placed at the left. Red nodes represent overexpressed miRs or genes; meanwhile, underexpressed molecules are depicted in blue. At the left side, networks constructed with overexpressed miRs and underexpressed genes can be found. The right part of the figures contains networks with underexpressed miRs and overexpressed genes. Green squares mark the location of miR-217, the only micro-RNA present in the four networks.

We conserved the top 100,000 miR–gene interactions for each one of the five networks (**Supplementary Material S3**). From the top 100,000 edge networks, we conserved only those miR–gene interactions between DEGs and DEMs with opposite differential expression trend (overexpressed miR, underexpressed gene, and *vice versa*). The resulting networks are depicted in **Figure 5**. The difference in size between the networks of *st_I_* and the rest of networks is evident.

### miR-217 Is Differentially Expressed in All Sequentially Contiguous Contrasts Yet Shows Different Target Genes for Each Stage

From **Figure 5**, it can be appreciated that, in each network, miR-217 appears as one of the DEMs and also has a target gene in all cases. In the contrast between stage I and NT, miR-217 is underexpressed (*Log_2_FC* = −1.32). In this stage, this micro-RNA potentially regulates up to 60 target genes (**Supplementary Material S4**). Among the target genes of miR-217, we can find BIRC7 (*Log_2_FC = 5.988*), LAMA4 (*Log_2_FC = 4.024*), or E2F2 (*Log_2_FC = 2.266*) genes (**Table 5**).

**Table 5:** Expression statistics for miR-217 and its target genes.

	NT–st_I_	st_I_–st_II_	st_II_–st_III_	st_III_–st_IV_
miR-217 *Log_2_FC*	-1.322	-0.5326	0.9033	-0.7065
Number of targets	60	GALNTL6	WNK2	IGF2BP2
Targets *Log_2_F*	1.899 (average)	1.1678	-1.2373	1.0798

For the contrast between stage I and NT, BIRC7 was the most overexpressed gene. BIRC7 has been reported to be crucial in the development of thyroid cancer by inhibiting apoptosis (49) in several cancer types, such as thyroid (50), leukemia (51), or neuroblastoma (52). In particular, for renal cell carcinoma, the overexpression of BIRC7 has been associated to PTEN-related malignancy and poorer prognosis (53) and metastatic behavior (54). **Supplementary Material S5** is a Cytoscape session file (a.cys network file) containing all top 100,000 networks as well as those for the differentially expressed miR–genes.

LAMA4 is also strongly overexpressed in stage I compared with NT. Its overexpression has been related305 to metastasis in pancreatic Cancer (55). Additionally, it has been observed that miR-200b306 down-regulated LAMA4 and decreases metastasis of renal cell carcinoma (56).

Regarding the stage II network, GALNTL6 (polypeptide N-acetylgalactosaminyltransferase like 6) is the only target of miR-217 present. This gene is related to the metabolism of proteins and O-linked glycosylation (57). The Gene Ontology (GO) annotations related to this gene include carbohydrate binding and polypeptide N-acetylgalactosaminyltransferase activity. GALNTs typically initiate O-glycosylation in the Golgi apparatus, but in cell culture models these enzymes can translocate to the ER *via* a process that involves aberrant Src signaling, leading to an increased density of O-glycosylation of MUC1 repeats (58). GALNTL6 has been reported to be amplified in papillary thyroid carcinomas (59).

For the stage III network, the only target of miR-217 is WNK2 (WNK lysine-deficient protein kinase 2). Diseases associated with WNK2 include hypomagnesemia 4 and renal and angiomatous meningioma. Pathways related to WNK2 are the transport of glucose and other sugars, bile salts and organic acids, and metal ions and amine compounds and ion channel transport. The GO annotations related to this gene include transferase activity, transferring phosphorus-containing groups, and protein tyrosine kinase activity.

We should notice that, in stage III network, WNK2 is underexpressed, and miR-217 is upregulated. WNK2 is considered as a tumor suppressor gene because it inhibits cell proliferation (60), negatively regulating epidermal growth factor receptor signaling *via* the inhibition of MEK1 (61).

Taking these issues into account, the fact that miR-217 resulted overexpressed and its only target in stage III network was WNK2 supports the hypothesis that WNK2 may be a stage-III-specific tumor suppressor gene downregulated by miR-217.

**Figure 6:**
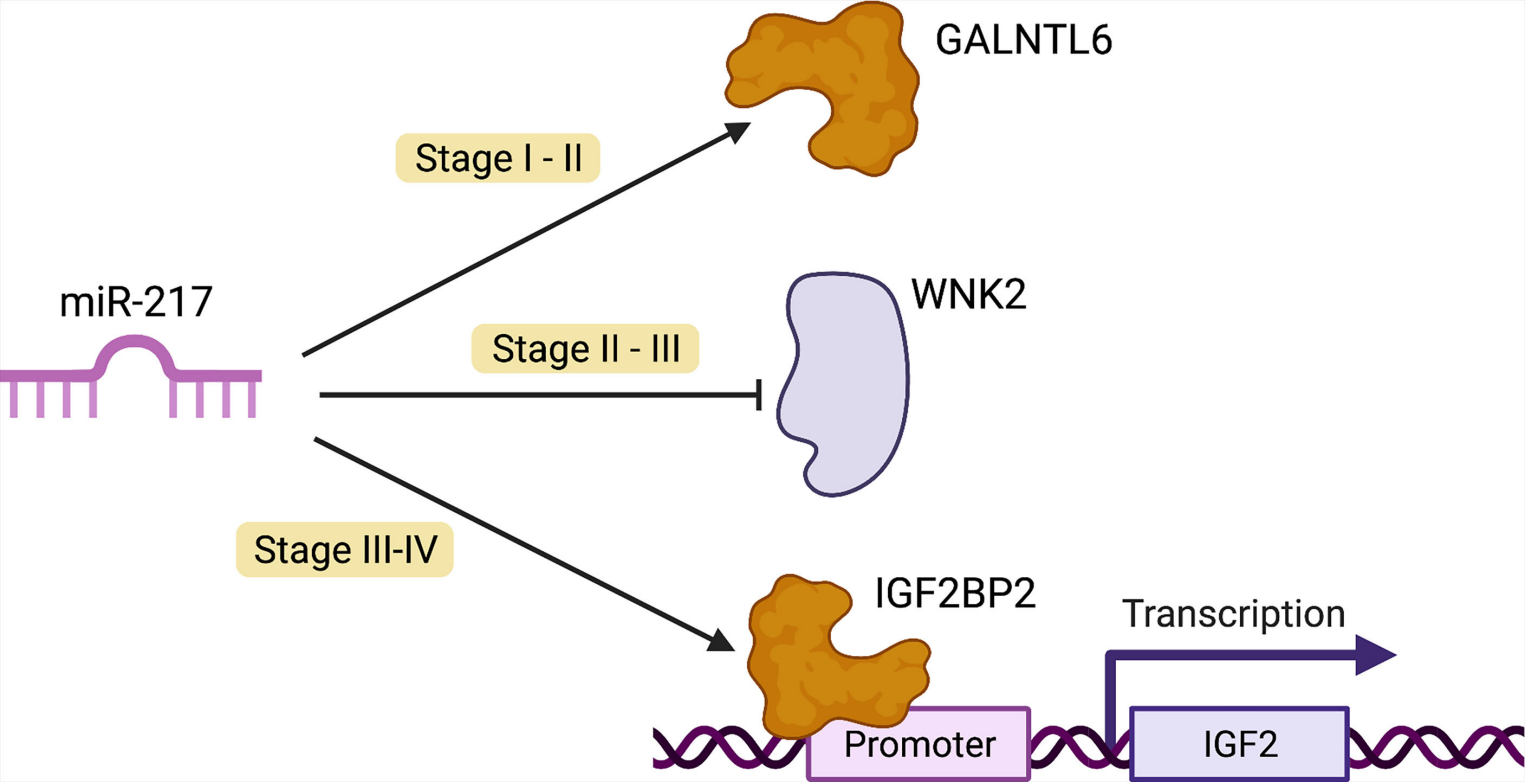
Possible oncogenic role of miR-217. In cancer transition one (stage I–II), miR-217 allows GALNTL6 overexpression. This protein typically initiates post-translational modifications in the Golgi apparatus. Additionally, in cell culture models, these enzymes affect the ER *via* aberrant Src signaling. In stages II–III (transition two), miR-217 represses WNK2 expression, a tumor suppressor which inhibits cell proliferation by negatively modulating the activation of the MEK1 pathway. In the last transition (stages III–IV), miR-217 enables IGF2BP2 overexpression. This gene promotes tumor progression in several types of cancer, such as glioblastoma multiforme and gallbladder cancer. IGF2BP2 also promotes tumor cell proliferation through the PI3K-Akt pathway (69).

Finally, we found IGF2BP2 as the unique target of miR-217 in the stage IV network. IGF2BP2 is an IGF2 (insulin growth factor 2) post-transcriptional regulator. Other targets of this gene are MYC and PTEN, two crucial participants in pathways associated with tumorigenesis (62). IGF2BP2 was considered as a metabolism regulator. It modulates cellular metabolism in diabetes, obesity, or fatty liver diseases by means of post-transcriptional gene regulation (63). Recently, it has been demonstrated that the overexpression of this gene is a prognostic factor in several types of cancer, such as leukemia (64), breast (65), lung (66), colorectal (67), or hepatocellular carcinoma (68) (**Figure 6**).

In the stage IV network, miR-217 is underexpressed, and its only target is IGF2BP2, which is overexpressed (*Log_2_FC* = 1.0798). The overexpression of this gene may be due to the underexpression of miR-217 in this stage of ccRC.

It is worth noticing that the differential expression of all aforementioned genes occurs between sequentially contiguous stages, *i*.*e*., the contrast between those genes is made by the previous phase of ccRC. These results are remarkable since the “control” dataset is an earlier stage of ccRC; that control gene expression dataset is already altered by cancer. Hence, DEGs and DEMs are “more differentiated” than in the control network, which is the traditionally selected contrast.

As shown in **Figures 2**, **3**, the number of statistically significant interactions in the *NT − st_I_* network is much larger than in any other contrast. This implies that the largest alterations occurring between these contiguous progression stages are given by the high number of differentially expressed genes and miRs, allowing the deregulation of several biological processes, which are, in turn, associated with radical changes of the whole phenotype.

On the other hand, the low number of interactions in the subsequent contrasts may also imply that the miR–gene deregulation observed in the advanced stages is a complementary process, which is concomitant to several other phenomena that drive the clear cell renal carcinoma progression.

We want to highlight an apparently counter-intuitive result; this is related to the number of shared differentially expressed genes/miRs in each tumor stage with respect to the shared interactions: the same set of 16,227 genes and 275 miRs was used to construct each network. However, as shown in **Figure 4A**, the number of shared interactions is very low compared with the unique interactions per network (more than 90,000 out of 100,000 for any given phenotype).

This effect of uniqueness in the network interactions most likely obeys the specificities of regulation by micro-RNAs in each context. Despite the fact that the five networks contain the same genes and miRs, the way in which miRs and genes co-express is exclusive. The progression of CCRC apparently modifies the micro-RNA-mediated genetic regulatory processes.

Notwithstanding, the network composed of the shared interactions between the four CCRC progression stages is also informative. From that network, we can observe that almost all interactions occur between genes and miRs from the same chromosome.

The bias to intra-chromosomal interactions has been previously reported by our group in gene co-expression networks for breast cancer (28, 48, 70–72), lung cancer (31), and also CCRC (32). These results show a clear trend to favor close gene correlations in terms of base pair distance. However, for miR–gene co-expression networks in breast cancer (14, 73, 74), we did not observe a trend to present more intra-chromosomal miR–gene interactions over inter-chromosomal ones. To our knowledge, this is the first time that a bias into the intra-chromosomal miR–gene interactions, in the context of breast cancer, was observed.

The finding of those 31 intra-chromosomal miR–gene interactions may be related to the same mechanism behind the bias favoring local correlations over the long-distance ones.

However, the mechanism for which this phenomenon emerges in cancer, but not in control, networks remains elusive. We have investigated the role of other biomolecular processes such as those in transcription factor binding sites, CTCF binding sites (30), or copy number alterations (75). It is worth noticing that none of them has shown to be significantly related to the loss of inter-chromosomal interactions.

Regarding the differences between progression stage networks, the low number of regulated genes by miRs is intriguing since the reports of genes targeted by miRs in the context of renal carcinoma has grown in the recent years [for a systematic review, see (76)]. The latter could be due to the form in which networks were constructed. These networks were obtained by three different filters: (a) the top 100,000 miR–gene co-expression interactions, (b) those miRs and genes that resulted differentially expressed between contiguous stages, and (c) the co-expression relationships between miRs and genes with opposite sign in their differential expression values.

## Concluding Remarks

In this work, we have constructed a set of networks in order to provide a framework for the evolution of the co-expression landscape of micro-RNAs and genes during the progression of clear cell renal carcinoma. As a summary of findings, we may establish the following:

- With this approach we were able to find differentially expressed genes and miRs for each progression stage. At the same time, we were capable of inferring networks filtered to look up for canonical miRs–gene regulatory interactions.
- The largest difference in terms of number of differentially expressed genes as well as in the number of miR–gene interactions occurring between control and stage I.
- Each network behaves differently in terms of miRs and genes involved. Those networks do not share interactions, and the large majority of miR–gene edges are indeed unique for each progression stage network.
- miR-217 is differentially expressed in all networks. It is the only micro-RNA that is differentially expressed in each stage with oppositely expressed gene targets.
- miR-217 correlates with a completely different set of genes depending on the progression stage. Furthermore, the differential expression of all those genes is in agreement with their role as oncogenes or tumor suppressor genes.
- The finding that LAMA4, BIRC7, GALNTL6, WNK2, and IGFBP2 are potential targets of miR-217 at different times of tumor evolution may help to develop stage-specific strategies, taking into account the differential expression of miR-217 in each stage of clear cell renal carcinoma progression.
- To our knowledge, this is the first time that the evolution of the expression patterns of a micro-RNA is tracked down during all steps of carcinoma progression and, at the same time, its ability to regulate different targets according to the tumor evolution is analyzed.

Possible extensions to the work presented here may include the analysis of other -omic sources, such as the methylation profile, the role of long non-coding RNAs, or the copy number alteration profile. The idea of integrating several sources to provide a more realistic model of the transcriptomic regulation in cancer is important for further steps towards an integrative understanding of gene regulatory programs in cancer.

Additional extensions could be related to the classification of samples based on other clinical features and not only in the progression stage, such as age, gender, or survival status.

A number of the hypotheses that this and other studies have generated must be experimentally tested under different conditions in order to fully capture the potential mechanisms and their implications. However, we believe that approaches such as this one could help the biomedical and clinical research in the search for stage-specific micro-RNA-targeted therapies.

## Data Availability Statement

The original contributions presented in the study are included in the article/**Supplementary Material**. Further inquiries can be directed to the corresponding author.

## Author Contributions

JZ-F performed the computational analyses, developed and implemented the programming code, performed pre-processing and low-level data analysis, made the figures, and drafted the manuscript. EH-L developed the theoretical approach, supervised the statistical analysis, designed the figures, and drafted and reviewed the manuscript. JE-E conceived and designed the project, supervised the project, made the figures, and drafted and reviewed the manuscript. All authors contributed to the article and approved the submitted version.

## Funding

This work was supported by CONACYT (267236 PhD student scholarship to JMZ-F) as well as by federal funding from the National Institute of Genomic Medicine (Mexico). JMZ-F is a doctoral student from the Programa de Doctorado en Ciencias Biomédicas, Universidad Nacional Autónoma de México. This work is part of his PhD thesis.

## Conflict of Interest

The authors declare that the research was conducted in the absence of any commercial or financial relationships that could be construed as a potential conflict of interest.

## Publisher’s Note

All claims expressed in this article are solely those of the authors and do not necessarily represent those of their affiliated organizations, or those of the publisher, the editors and the reviewers. Any product that may be evaluated in this article, or claim that may be made by its manufacturer, is not guaranteed or endorsed by the publisher.

## Supplementary Material

Supplementary Material S1 Venn diagrams of genes and micro-RNAs crossing all elements in contrast non-tumor stages. This zip file contains six Venn diagrams for upregulated, downregulated, and all elements according to 2.4.

Supplementary Material S2 Quality control and normalization results for gene expression in the five phenotypes. This zip file contains PCA plots before and after normalization for genes and micro-RNAs. All plots were performed after applying filters reported in 2.3.

Supplementary Material S3 Differential expression values for all combinations between four stages of ccRC and non-tumor. This zip file contains 20 differential expression analyses, including log_2_ fold change value, *p*-value, adjusted *p*-value, and *B*-statistic for each gene. BioMart annotation in every gene is included for better use.

Supplementary Material S4 Targets of miR217. The table contains log_2_ fold change value and *p*-value. Additionally, each gene was annotated with BioMart data.

Supplementary Material S5 Networks processed. The compressed file contains a.*cys* file with all networks described in this work. The networks are merged with differential expression data. All networks were classified by stage, contrast, and log_2_ fold change.

Supplementary Material S6 Molecular features of microRNAs and genes of the 33 cancer-shared interactions.

## References

[1] SEER. Cancer Stat Facts: Kidney and Renal Pelvis Cancer. Available at: https://seer.cancer.gov/statfacts/html/kidrp.html (Accessed January, 2022).

[2] Haas NB Nathanson KL. Hereditary Kidney Cancer Syndromes. Adv Chronic Kidney Dis (2014) 21:81–90. doi: 10.1053/j.ackd.2013.10.001 24359990 PMC3872053

[3] Weidle UH Nopora A. Clear Cell Renal Carcinoma: MicroRNAs With Efficacy in Preclinical *In Vivo* Models. Cancer Genomics - Proteomics (2021) 18:349–68. doi: 10.21873/cgp.20265 PMC8240043 33994361

[4] Jonasch E Walker CL Rathmell WK. Clear Cell Renal Cell Carcinoma Ontogeny and Mechanisms of Lethality. Nat Rev Nephrol (2020) 17:245–61. doi: 10.1038/s41581-020-00359-2 PMC8172121 33144689

[5] Hsieh JJ Purdue MP Signoretti S Swanton C Albiges L Schmidinger M. Renal Cell Carcinoma. Nat Rev Dis Primers (2017) 3:1–19. doi: 10.1038/nrdp.2017.9 PMC5936048 28276433

[6] Chevrier S Levine JH Zanotelli VRT Silina K Schulz D Bacac M. An Immune Atlas of Clear Cell Renal Cell Carcinoma. Cell (2017) 169:736–749.e18. doi: 10.1016/j.cell.2017.04.016 28475899 PMC5422211

[7] Wang T Lu R Kapur P Jaiswal BS Hannan R Zhang Z. An Empirical Approach Leveraging Tumorgrafts to Dissect the Tumor Microenvironment in Renal Cell Carcinoma Identifies Missing Link to Prognostic Inflammatory Factors. Cancer Discov (2018) 8:1142–55. doi: 10.1158/2159-8290.cd-17-1246 PMC6125163 29884728

[8] Liu XD Kong W Peterson CB McGrail DJ Hoang A Zhang X. PBRM1 Loss Defines a Nonimmunogenic Tumor Phenotype Associated With Checkpoint Inhibitor Resistance in Renal Carcinoma. Nat Commun (2020) 11. doi: 10.1038/s41467-020-15959-6 PMC7195420 32358509

[9] Friedman RC Farh KKH Burge CB Bartel DP. Most Mammalian Mrnas are Conserved Targets of Micrornas. Genome Res (2009) 19:92–105. doi: 10.1101/gr.082701.108 18955434 PMC2612969

[10] Li M Marin-Muller C Bharadwaj U Chow KH Yao Q Chen C. Micrornas: Control and Loss of Control in Human Physiology and Disease. World J Surg (2009) 33:667–84. doi: 10.1007/s00268-008-9836-x PMC2933043 19030926

[11] Abd-Aziz N Kamaruzman NI Poh CL. Development of MicroRNAs as Potential Therapeutics Against Cancer. J Oncol (2020) 2020:1–14. doi: 10.1155/2020/8029721 PMC7378626 32733559

[12] Xu M Gu M Zhang K Zhou J Wang Z Da J. miR-203 Inhibition of Renal Cancer Cell Proliferation, Migration and Invasion by Targeting of FGF2. Diagn Pathol (2015) 10:24. doi: 10.1186/s13000-015-0255-7 25890121 PMC4419389

[13] Olson P Lu J Zhang H Shai A Chun MG Wang Y. MicroRNA Dynamics in the Stages of Tumorigenesis Correlate With Hallmark Capabilities of Cancer. Genes Dev (2009) 23:2152–65. doi: 10.1101/gad.1820109 PMC2751988 19759263

[14] Drago-García D Espinal-Enríquez J Hernández-Lemus E. Network Analysis of EMT and MET Micro-RNA Regulation in Breast Cancer. Sci Rep (2017) 7:1–17. doi: 10.1038/s41598-017-13903-1 PMC5648819 29051564

[15] O'Brien J Hayder H Zayed Y Peng C. Overview of MicroRNA Biogenesis, Mechanisms of Actions, and Circulation. Front Endochrinology (2018) 9:402. doi: 10.3389/fendo.2018.00402 PMC6085463 30123182

[16] Hashimoto Y Akiyama Y Yuasa Y. Multiple-To-Multiple Relationships Between Micrornas and Target Genes in Gastric Cancer. PloS One (2013) 8:e62589. doi: 10.1371/journal.pone.0062589 23667495 PMC3648557

[17] Di Leva G Garofalo M Croce CM. Micrornas in Cancer. Annu Rev Pathology: Mech Dis (2014) 9:287–314. doi: 10.1146/annurev-pathol-012513-104715 PMC4009396 24079833

[18] Garzon R Calin GA Croce CM. Micrornas in Cancer. Annu Rev Med (2009) 60:167–79. doi: 10.1146/annurev.med.59.053006.104707 19630570

[19] Hayes J Peruzzi PP Lawler S. Micrornas in Cancer: Biomarkers, Functions and Therapy. Trends Mol Med (2014) 20:460–9. doi: 10.1016/j.molmed.2014.06.005 25027972

[20] Han N Li H Wang H. Microrna-203 Inhibits Epithelial-Mesenchymal Transition, Migration, and Invasion of Renal Cell Carcinoma Cells *via* the Inactivation of the Pi3k/Akt Signaling Pathway by Inhibiting Cav1. Cell Adhesion Migration (2020) 14:227–41. doi: 10.1080/19336918.2020.1827665 PMC7714454 32990143

[21] Amar D Safer H Shamir R. Dissection of Regulatory Networks That are Altered in Disease *via* Differential Co-Expression. PloS Comput Biol (2013) 9:e1002955. doi: 10.1371/journal.pcbi.1002955 23505361 PMC3591264

[22] Alcalá-Corona SA de Anda-Jáuregui G Espinal-Enríquez J Hernández-Lemus E. Network Modularity in Breast Cancer Molecular Subtypes. Front Physiol (2017) 8:915. doi: 10.3389/fphys.2017.00915 29204123 PMC5699328

[23] van Dam S Vosa U van der Graaf A Franke L de Magalhaes JP. Gene Co-Expression Analysis for Functional Classification and Gene–Disease Predictions. Briefings Bioinf (2018) 19:575–92. doi: 10.1093/bib/bbw139 PMC6054162 28077403

[24] Fionda V. Networks in Biology. In: Ranganathan S Gribskov M Nakai K Schönbach C, editors. Encyclopedia of Bioinformatics and Computational Biology. Oxford: Academic Press (2019). p. 915 – 921. Available at: 10.1016/B978-0-12-809633-8.20420-2.

[25] Tieri P Farina L Petti M Astolfi L Paci P Castiglione F. Network Inference and Reconstruction in Bioinformatics. In: Encyclopedia of Bioinformatics and Computational Biology. Amsterdam: Elsevier (2019).

[26] Hassler MR Egger G. Epigenomics of Cancer – Emerging New Concepts. Biochimie (2012) 94:2219–30. doi: 10.1016/j.biochi.2012.05.007 PMC3480634 22609632

[27] Margolin AA Nemenman I Basso K Wiggins C Stolovitzky G Dalla Favera R. Aracne: An Algorithm for the Reconstruction of Gene Regulatory Networks in a Mammalian Cellular Context. BMC Bioinf (2006) 7:1–15. doi: 10.1186/1471-2105-7-S1-S7 PMC1810318 16723010

[28] Espinal-Enriquez J Fresno C Anda-Jáuregui G Hernández-Lemus E. Rna-Seq Based Genome-Wide Analysis Reveals Loss of Inter-Chromosomal Regulation in Breast Cancer. Sci Rep (2017) 7:1–19. doi: 10.1038/s41598-017-01314-1 28496157 PMC5431987

[29] de Anda-Jáuregui G Fresno C García-Cortés D Espinal-Enríquez J Hernández-Lemus E. Intrachromosomal Regulation Decay in Breast Cancer. Appl Mathematics Nonlinear Sci (2019) 4:223–30. doi: 10.2478/AMNS.2019.1.00020

[30] García-Cortés D de Anda-Jáuregui G Fresno C Hernandez-Lemus E Espinal-Enriquez J. Gene Co-Expression is Distance-Dependent in Breast Cancer. Front Oncol (2020) 10:1232. doi: 10.3389/fonc.2020.01232. 32850369 PMC7396632

[31] Andonegui-Elguera SD Zamora-Fuentes JM Espinal-Enríquez J Hernández-Lemus E. Loss of Long Distance Co-Expression in Lung Cancer. Front Genet (2021) 12:625741. doi: 10.3389/fgene.2021.625741 33777098 PMC7987938

[32] Zamora-Fuentes JM Hernández-Lemus E Espinal-Enríquez J. Gene Expression and Co-Expression Networks are Strongly Altered Through Stages in Clear Cell Renal Carcinoma. Front Genet (2020) 11:578679. doi: 10.3389/fgene.2020.578679 33240325 PMC7669746

[33] Creighton CJ Morgan M Gunaratne PH Wheeler DA Gibbs RA. Comprehensive Molecular Characterization of Clear Cell Renal Cell Carcinoma. Nature (2013) 499:43. doi: 10.1038/nature12222 23792563 PMC3771322

[34] Network CGAR. Comprehensive Molecular Characterization of Papillary Renal-Cell Carcinoma. N Engl J Med (2016) 374:135–45. doi: 10.1056/NEJMoa1505917 PMC4775252 26536169

[35] Ricketts CJ De Cubas AA Fan H Smith CC Lang M Reznik E. The Cancer Genome Atlas Comprehensive Molecular Characterization of Renal Cell Carcinoma. Cell Rep (2018) 23:313–26. doi: 10.1016/j.celrep.2018.03.075 PMC6075733 29617669

[36] Risso D Schwartz K Sherlock G Dudoit S. Gc-Content Normalization for Rna-Seq Data. BMC Bioinf (2011) 12:1–17. doi: 10.1186/1471-2105-12-480 PMC3315510 22177264

[37] Nueda Mj Ferrer A Conesa A. Arsyn: A Method for the Identification and Removal of Systematic Noise in Multifactorial Time Course Microarray Experiments. Biostatistics (2012) 13:553–66. doi: 10.1093/biostatistics/kxr042 22085896

[38] Love MI Huber W Anders S. Moderated Estimation of Fold Change and Dispersion for RNA-Seq Data With Deseq2. Genome Biol (2014) 15:1–21. doi: 10.1186/s13059-014-0550-8 PMC4302049 25516281

[39] Shannon P Markiel A Ozier O Baliga NS Wang JT Ramage D. Cytoscape: A Software Environment for Integrated Models of Biomolecular Interaction Networks. Genome Res (2003) 13:2498–504. doi: 10.1101/gr.1239303 PMC403769 14597658

[40] Kong W He L Richards EJ Challa S Xu CX Permuth-Wey J. Upregulation of miRNA-155 Promotes Tumour Angiogenesis by Targeting VHL and is Associated With Poor Prognosis and Triple-Negative Breast Cancer. Oncogene (2013) 33:679–89. doi: 10.1038/onc.2012.636 PMC3925335 23353819

[41] Neal CS Michael MZ Rawlings LH der Hoek MBV Gleadle JM. The VHL-Dependent Regulation of microRNAs in Renal Cancer. BMC Med (2010) 8:1–17. doi: 10.1186/1741-7015-8-64 PMC2978113 20964835

[42] Shah N Sukumar S. The Hox Genes and Their Roles in Oncogenesis. Nat Rev Cancer (2010) 10:361–71. doi: 10.1038/nrc2826 20357775

[43] Bhatlekar S Fields JZ Boman BM. Hox Genes and Their Role in the Development of Human Cancers. J Mol Med (2014) 92:811–23. doi: 10.1007/s00109-014-1181-y 24996520

[44] Li B Huang Q Wei GH. The Role of Hox Transcription Factors in Cancer Predisposition and Progression. Cancers (2019) 11:528. doi: 10.3390/cancers11040528 PMC6520925 31013831

[45] Meyer SE Muench DE Rogers AM Newkold TJ Orr E O’Brien E. Mir-196b Target Screen Reveals Mechanisms Maintaining Leukemia Stemness With Therapeutic Potential. J Exp Med (2018) 215:2115–36. doi: 10.1084/jem.20171312 PMC6080909 29997117

[46] Rawat VP Götze M Rasalkar A Vegi NM Ihme S Thoene S. The Microrna Mir-196b Acts as a Tumor Suppressor in Cdx2-Driven Acute Myeloid Leukemia. Haematologica (2020) 105:e285. doi: 10.3324/haematol.2019.223297 31558674 PMC7271585

[47] Xu F Zhu F Wang W Gao W Chen X Yu C. Down-Regulation of Mirna-196b Expression Inhibits the Proliferation, Migration and Invasiveness of Hepg2 Cells While Promoting Their Apoptosis *via* the Pi3k/Akt Signaling Pathway. Cell Mol Biol (2020) 66:159–64. doi: 10.14715/cmb/2020.66.3.25 32538764

[48] García-Cortés D Hernández-Lemus E Espinal-Enríquez J. Luminal a Breast Cancer Co-Expression Network: Structural and Functional Alterations. Front Genet (2021) 12. doi: 10.3389/fgene.2021.629475 PMC8096206 33959148

[49] Rigato DB Branco PC Mateus Reis Silva CS Machado-Neto JA Costa-Lotufo LV Jimenez PC. Birc7 (Baculoviral Iap Repeat Containing 7). Atlas Genet Cytogenetics Oncol Haematology.

[50] Liu K Yu Q Li H Xie C Wu Y Ma D. Birc7 Promotes Epithelial-Mesenchymal Transition and Metastasis in Papillary Thyroid Carcinoma Through Restraining Autophagy. Am J Cancer Res (2020) 10:78. 32064154 PMC7017743

[51] Ibrahim L Aladle D Mansour A Hammad A Al Wakeel AA Abd El-Hameed SA. Expression and Prognostic Significance of Livin/Birc7 in Childhood Acute Lymphoblastic Leukemia. Med Oncol (2014) 31:1–8. doi: 10.1007/s12032-014-0941-4 24696218

[52] Dasgupta A Alvarado CS Xu Z Findley HW. Expression and Functional Role of Inhibitor-of-Apoptosis Protein Livin (Birc7) in Neuroblastoma. Biochem Biophys Res Commun (2010) 400:53–9. doi: 10.1016/j.bbrc.2010.08.001 20691667

[53] Cheng T Zhang JG Cheng YH Gao ZW Ren XQ. Relationship Between Pten and Livin Expression and Malignancy of Renal Cell Carcinomas. Asian Pacific J Cancer Prev (2012) 13:2681–5. doi: 10.7314/APJCP.2012.13.6.2681 22938441

[54] Wagener N Crnković-Mertens I Vetter C Macher-Göppinger S Bedke J Gröne E. Expression of Inhibitor of Apoptosis Protein Livin in Renal Cell Carcinoma and non-Tumorous Adult Kidney. Br J Cancer (2007) 97:1271–6. doi: 10.1038/sj.bjc.6604028 PMC2360474 17968430

[55] Zheng B Qu J Ohuchida K Feng H Chong SJF Yan Z. Lama4 Upregulation is Associated With High Liver Metastasis Potential and Poor Survival Outcome of Pancreatic Cancer. Theranostics (2020) 10:10274. doi: 10.7150/thno.47001 32929348 PMC7481422

[56] Li Y Guan B Liu J Zhang Z He S Zhan Y. Microrna-200b is Downregulated and Suppresses Metastasis by Targeting Lama4 in Renal Cell Carcinoma. EBioMedicine (2019) 44:439–51. doi: 10.1016/j.ebiom.2019.05.041 PMC6604878 31130475

[57] Bateman A Martin MJ Orchard S Magrane M Agivetova R Ahmad S. Uniprot: The Universal Protein Knowledgebase in 2021. Nucleic Acids Res (2020) 49:D480–ndash;9. doi: 10.1093/nar/gkaa1100 PMC7778908 33237286

[58] Reily C Stewart TJ Renfrow MB Novak J. Glycosylation in Health and Disease. Nat Rev Nephrol (2019) 15:346–66. doi: 10.1038/s41581-019-0129-4 PMC6590709 30858582

[59] Passon N Bregant E Sponziello M Dima M Rosignolo F Durante C. Somatic Amplifications and Deletions in Genome of Papillary Thyroid Carcinomas. Endocrine (2015) 50:453–64. doi: 10.1007/s12020-015-0592-z 25863487

[60] Moniz S Verissimo F Matos P Brazao R Silva E Kotevelets L. Protein Kinase Wnk2 Inhibits Cell Proliferation by Negatively Modulating the Activation of Mek1/Erk1/2. Oncogene (2007) 26:6071–81. doi: 10.1038/sj.onc.1210706 17667937

[61] Jun P Hong C Lal A Wong JM McDermott MW Bollen AW. Epigenetic Silencing of the Kinase Tumor Suppressor Wnk2 is Tumor-Type and Tumor-Grade Specific. Neuro-oncology (2009) 11:414–22. doi: 10.1215/15228517-2008-096 PMC2743221 19001526

[62] Bell JL Wächter K Mühleck B Pazaitis N Köhn M Lederer M. Insulin-Like Growth Factor 2 mRNA-Binding Proteins (IGF2bps): Post-Transcriptional Drivers of Cancer Progression? Cell Mol Life Sci (2012) 70:2657–75. doi: 10.1007/s00018-012-1186-z PMC3708292 23069990

[63] Wang J Chen L Qiang P. The Role of Igf2bp2, an M6a Reader Gene, in Human Metabolic Diseases and Cancers. Cancer Cell Int (2021) 21:1–11. doi: 10.1186/s12935-021-01799-x 33568150 PMC7876817

[64] He X Li W Liang X Zhu X Zhang L Huang Y. Igf2bp2 Overexpression Indicates Poor Survival in Patients With Acute Myelocytic Leukemia. Cell Physiol Biochem (2018) 51:1945–56. doi: 10.1159/000495719 30513526

[65] Li X Li Y Lu H. Mir-1193 Suppresses Proliferation and Invasion of Human Breast Cancer Cells Through Directly Targeting Igf2bp2. Oncol Res Featuring Preclinical Clin Cancer Ther (2017) 25:579–85. doi: 10.3727/97818823455816X14760504645779 PMC7841109 27733218

[66] Huang Rs Zheng Yl Li C Ding C Xu C Zhao J. Microrna-485-5p Suppresses Growth and Metastasis in non-Small Cell Lung Cancer Cells by Targeting Igf2bp2. Life Sci (2018) 199:104–11. doi: 10.1016/j.lfs.2018.03.005 29510198

[67] Ye S Song W Xu X Zhao X Yang L. Igf2bp2 Promotes Colorectal Cancer Cell Proliferation and Survival Through Interfering With Raf-1 Degradation by Mir-195. FEBS Lett (2016) 590:1641–50. doi: 10.1002/1873-3468.12205 27153315

[68] Wei H Pu J Wang J Chuan W Xu Z Wu X. Igf2bp2 Promotes Liver Cancer Growth Through an M6a-Fen1-Dependent Mechanism. Front Oncol (2020) 10:2377. doi: 10.3389/fonc.2020.578816 PMC7667992 33224879

[69] Xu X Yu Y Zong K Lv P Gu Y. Up-Regulation of Igf2bp2 by Multiple Mechanisms in Pancreatic Cancer Promotes Cancer Proliferation by Activating the Pi3k/Akt Signaling Pathway. J Exp Clin Cancer Res (2019) 38:497. doi: 10.1186/s13046-019-1470-y 31852504 PMC6921559

[70] Dorantes-Gilardi R García-Cortés D Hernández-Lemus E Espinal-Enríquez J. Multilayer Approach Reveals Organizational Principles Disrupted in Breast Cancer Co-Expression Networks. Appl Network Sci (2020) 5:1–23. doi: 10.1007/s41109-020-00291-1

[71] González-Espinoza A Zamora J Hernandez-Lemus E Espinal-Enríquez J. Gene Co-Expression in Breast Cancer: A Matter of Distance. Front Oncol (2021) 1:4743. doi: 10.3389/fonc.2021.726493 PMC8636045 34868919

[72] Dorantes-Gilardi R García-Cortés D Hernández-Lemus E Espinal-Enríquez J. K-Core Genes Underpin Structural Features of Breast Cancer. Sci Rep (2021) 11:1–17. doi: 10.1038/s41598-021-95313-y 34381069 PMC8358063

[73] de Anda-Jáuregui G Espinal-Enríquez J Drago-García D Hernández-Lemus E. Nonredundant, Highly Connected Micrornas Control Functionality in Breast Cancer Networks. Int J Genomics (2018) 2018:1–11. doi: 10.1155/2018/9585383 PMC5996465 30003085

[74] de Anda-Jáuregui G Espinal-Enríquez J Hernández-Lemus E. Highly Connected, non-Redundant Microrna Functional Control in Breast Cancer Molecular Subtypes. Interface Focus (2021) 11:20200073. doi: 10.1098/rsfs.2020.0073 34123357 PMC8193465

[75] Hernández-Gómez C Hernández-Lemus E Espinal-Enríquez J. The Role of Copy Number Variants in Gene Co-Expression Patterns for Luminal B Breast Tumors. Front Genet (2022) 13:806607–7. doi: 10.3389/fgene.2022.806607 PMC9010943 35432489

[76] Li M Wang Y Song Y Bu R Yin B Fei X. Micrornas in Renal Cell Carcinoma: A Systematic Review of Clinical Implications. Oncol Rep (2015) 33:1571–8. doi: 10.3892/or.2015.3799 PMC4358077 25682771

