# Supplementary figures and images for: Oncogenic Role of miR-217 During Clear Cell Renal Carcinoma Progression
Source: Front Oncol. 2022 Jul 22;12:934711. doi: 10.3389/fonc.2022.934711 (PMC9354686; doi:10.3389/fonc.2022.934711)

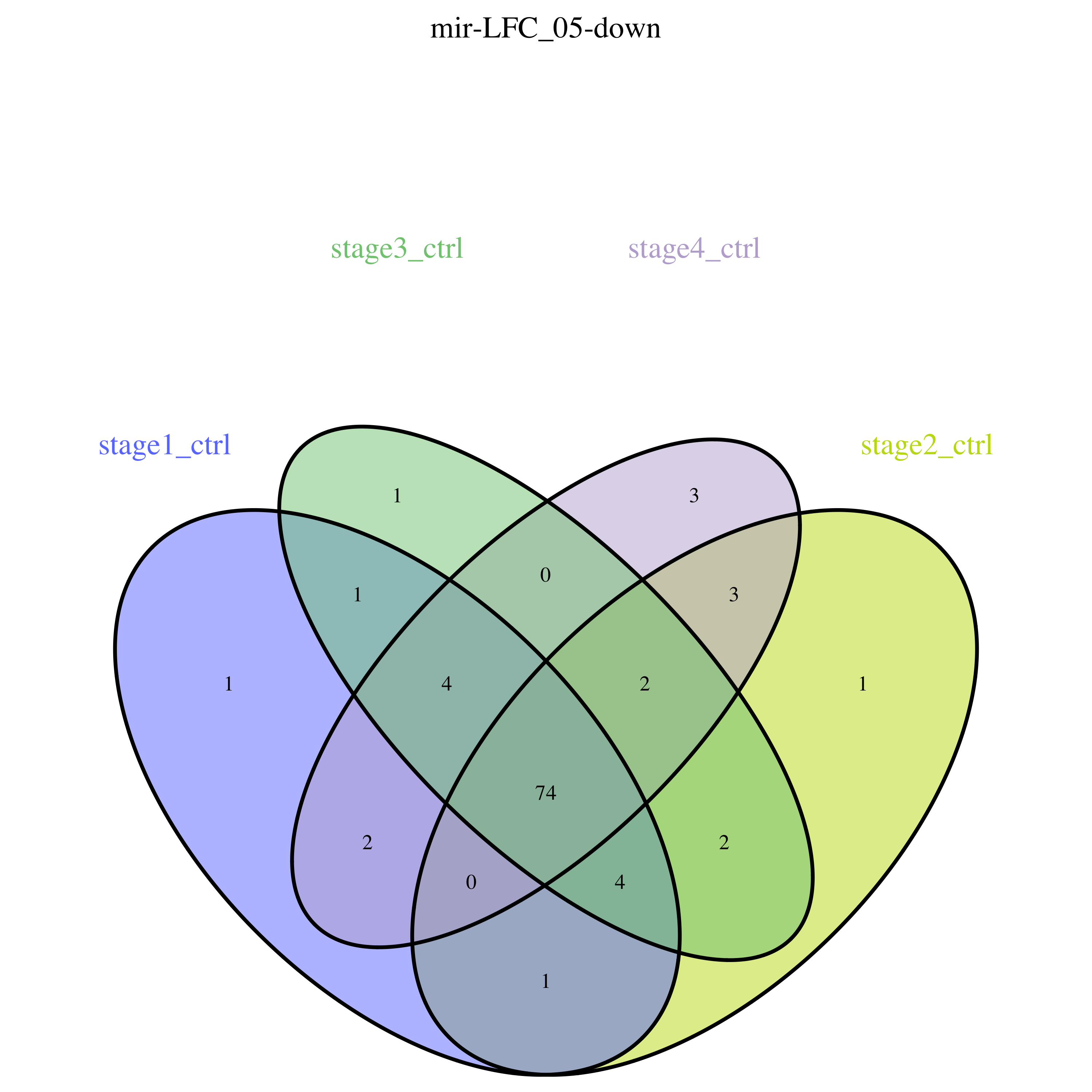

Supplement: Supplementary Material S1 — Venn diagrams of genes and micro-RNAs crossing all elements in contrast non-tumor stages. This zip file contains six Venn diagrams for upregulated, downregulated, and all elements according to 2.4. [file DataSheet_1.zip › venn-mirna-gene/venn-mir-LFC_05-down (1).png]

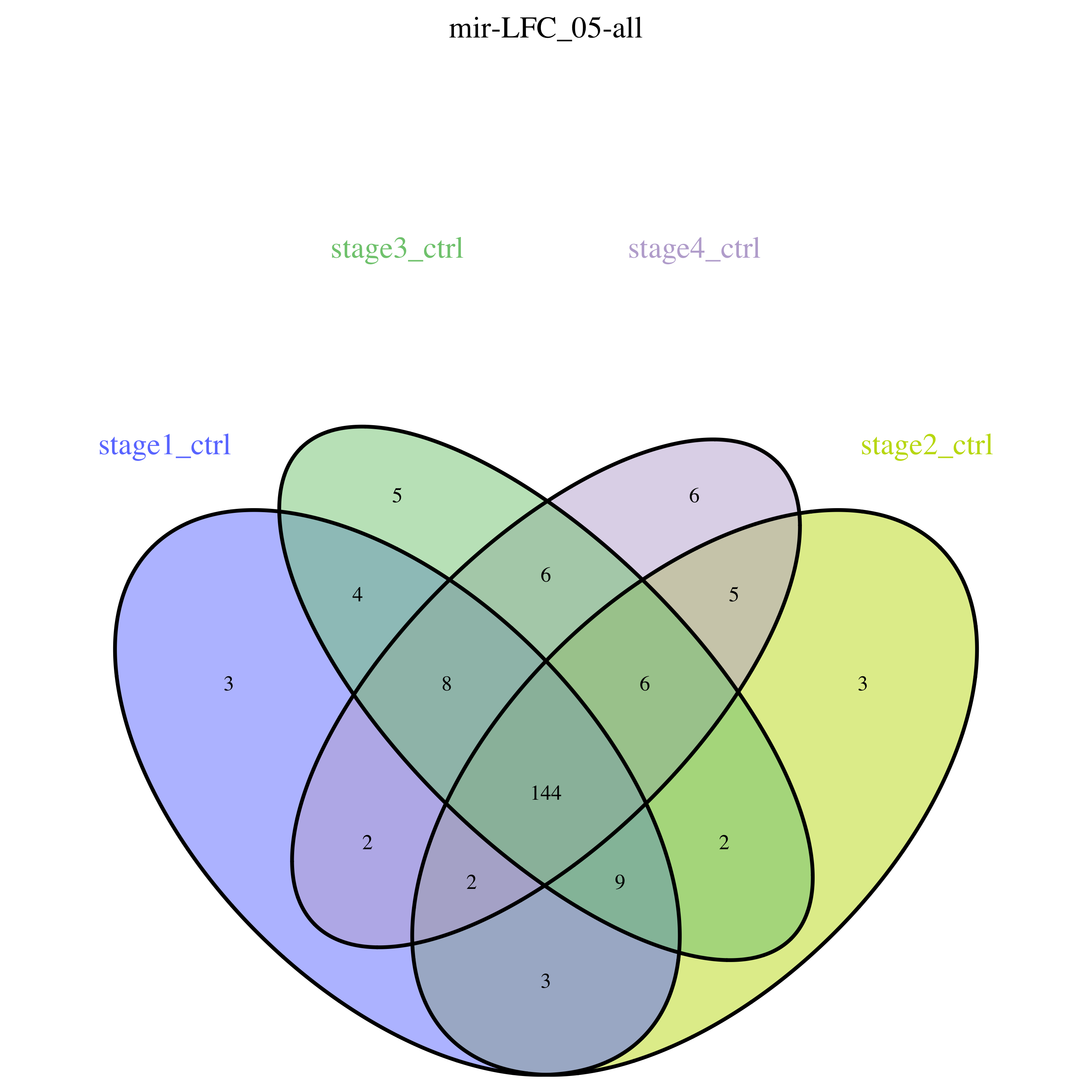

Supplement: Supplementary Material S1 — Venn diagrams of genes and micro-RNAs crossing all elements in contrast non-tumor stages. This zip file contains six Venn diagrams for upregulated, downregulated, and all elements according to 2.4. [file DataSheet_1.zip › venn-mirna-gene/venn-mir-LFC_05-all (1).png]

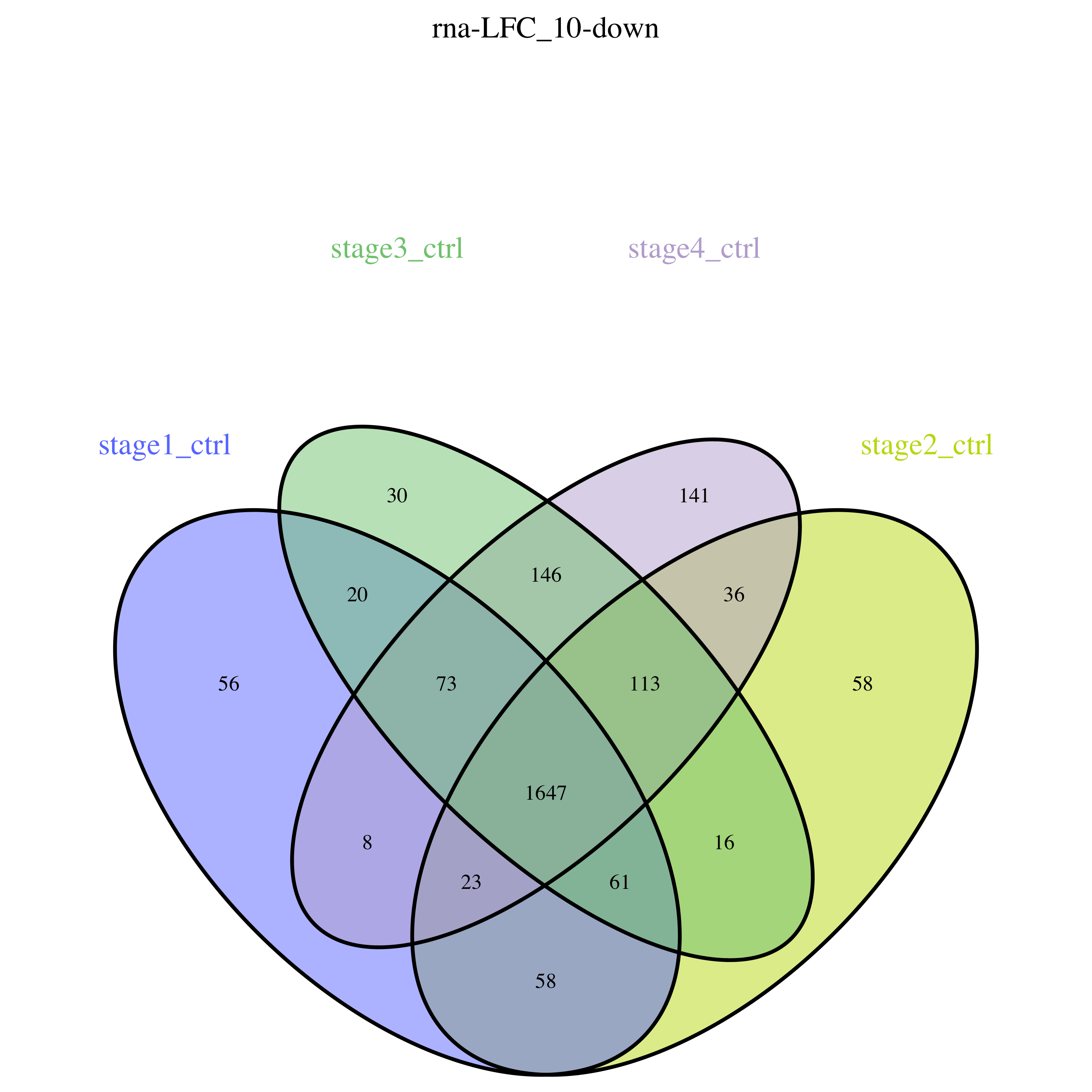

Supplement: Supplementary Material S1 — Venn diagrams of genes and micro-RNAs crossing all elements in contrast non-tumor stages. This zip file contains six Venn diagrams for upregulated, downregulated, and all elements according to 2.4. [file DataSheet_1.zip › venn-mirna-gene/venn-rna-LFC_10-down.png]

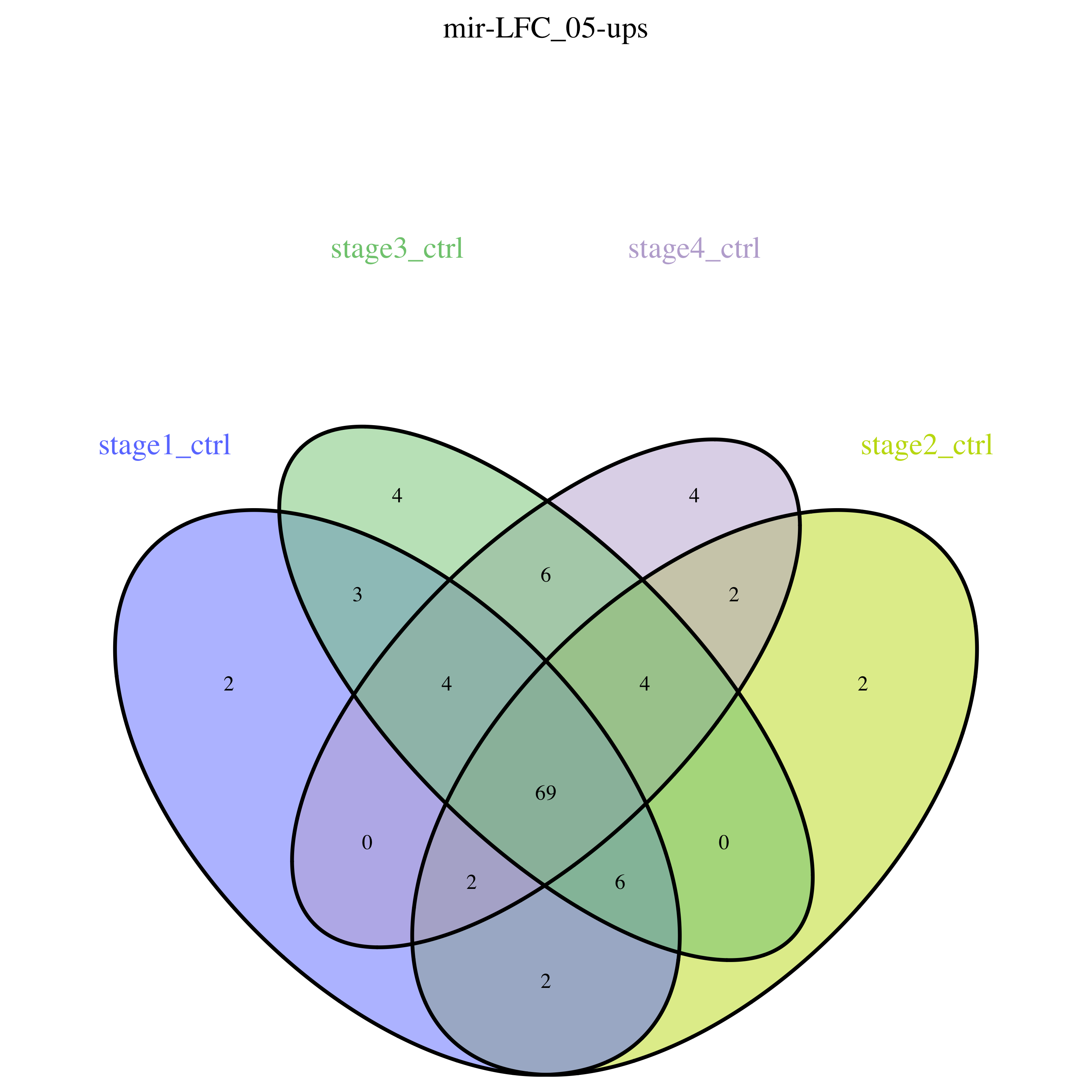

Supplement: Supplementary Material S1 — Venn diagrams of genes and micro-RNAs crossing all elements in contrast non-tumor stages. This zip file contains six Venn diagrams for upregulated, downregulated, and all elements according to 2.4. [file DataSheet_1.zip › venn-mirna-gene/venn-mir-LFC_05-ups.png]

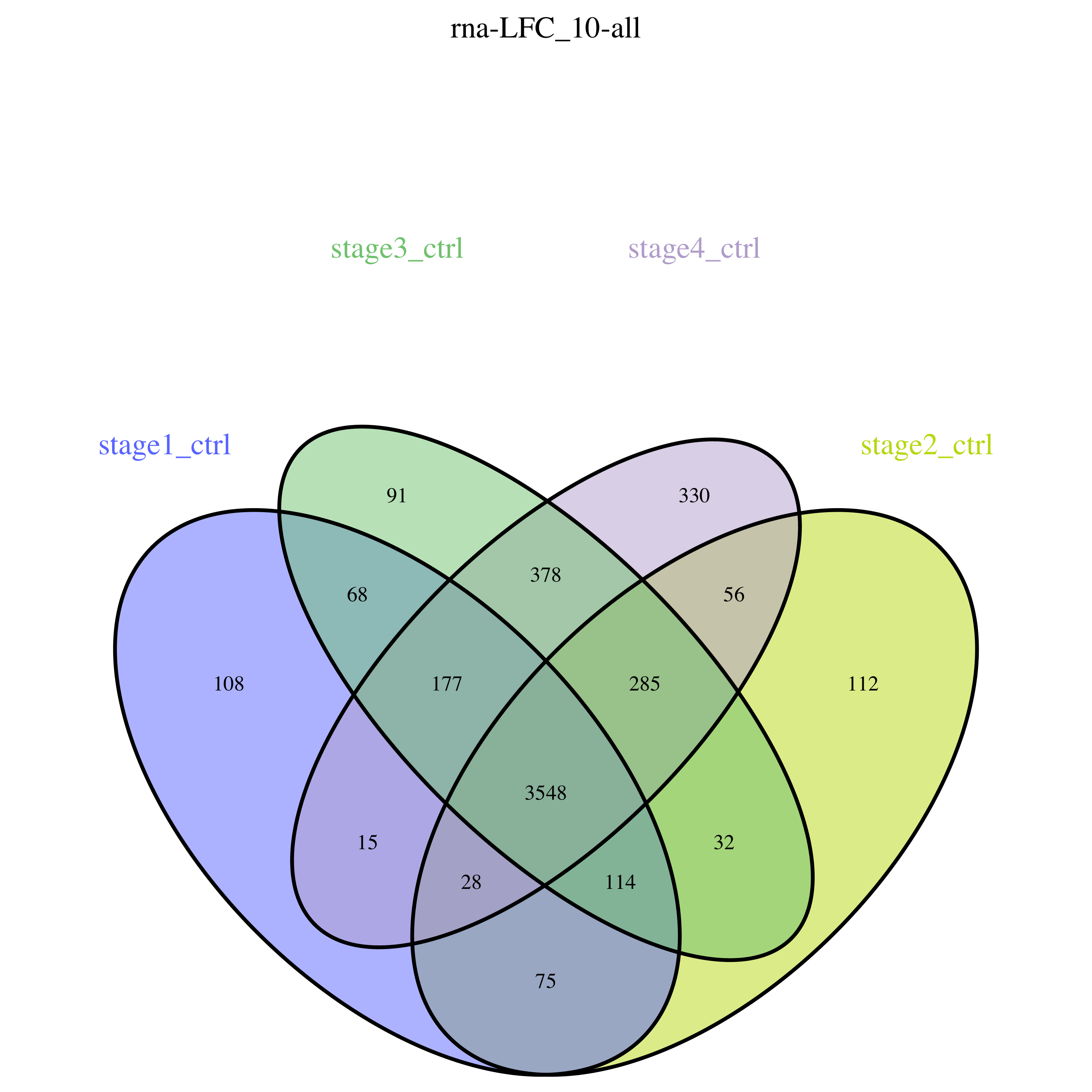

Supplement: Supplementary Material S1 — Venn diagrams of genes and micro-RNAs crossing all elements in contrast non-tumor stages. This zip file contains six Venn diagrams for upregulated, downregulated, and all elements according to 2.4. [file DataSheet_1.zip › venn-mirna-gene/venn-rna-LFC_10-all (1).png]

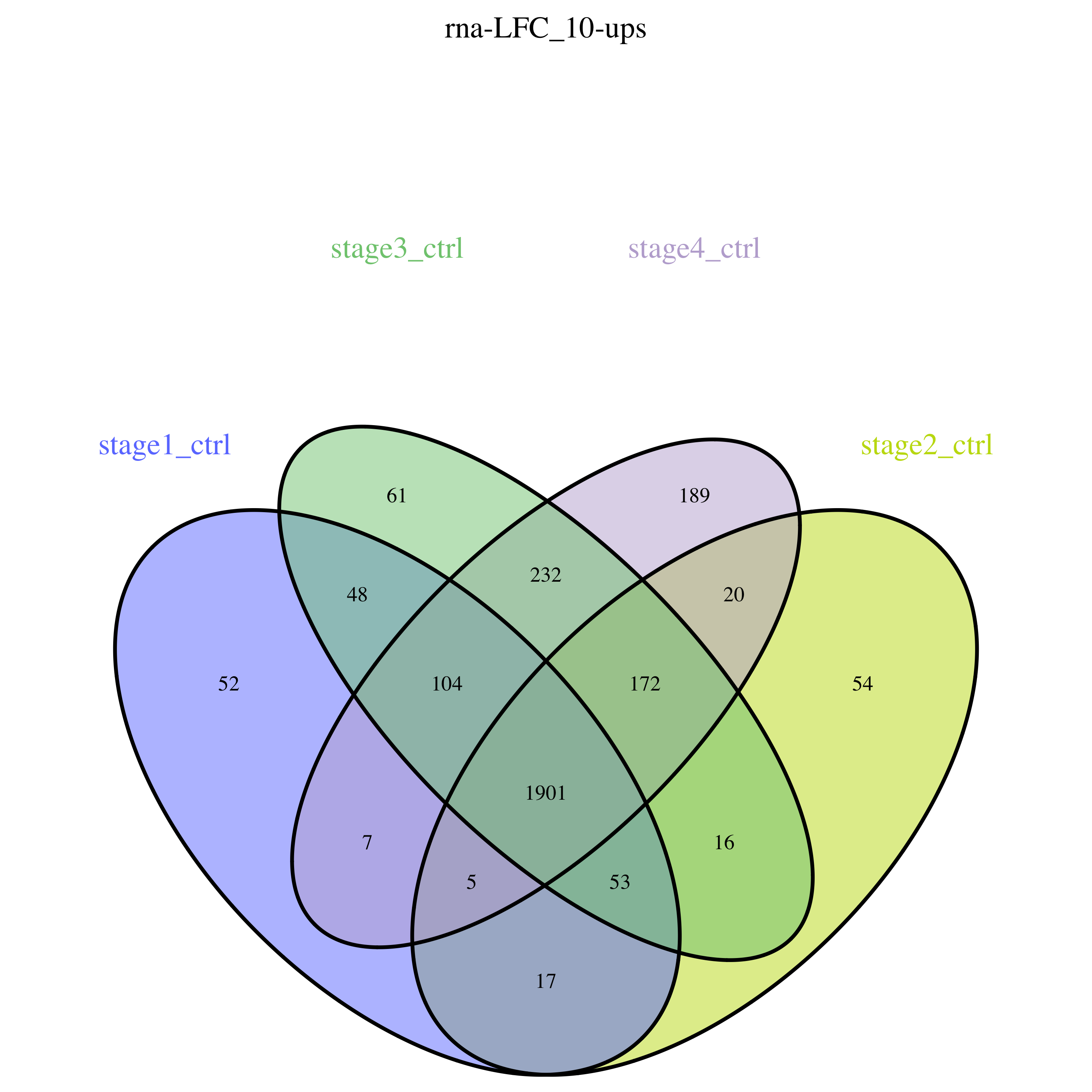

Supplement: Supplementary Material S1 — Venn diagrams of genes and micro-RNAs crossing all elements in contrast non-tumor stages. This zip file contains six Venn diagrams for upregulated, downregulated, and all elements according to 2.4. [file DataSheet_1.zip › venn-mirna-gene/venn-rna-LFC_10-ups.png]

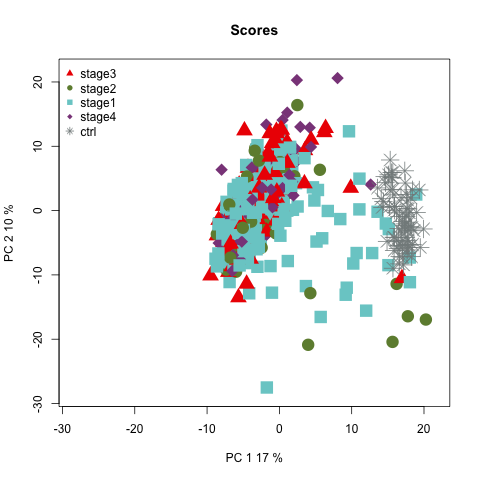

Supplement: Supplementary Material S2 — Quality control and normalization results for gene expression in the five phenotypes. This zip file contains PCA plots before and after normalization for genes and micro-RNAs. All plots were performed after applying filters reported in 2.3. [file DataSheet_2.zip › QC/PCA-mir-BeforeNorm.png]

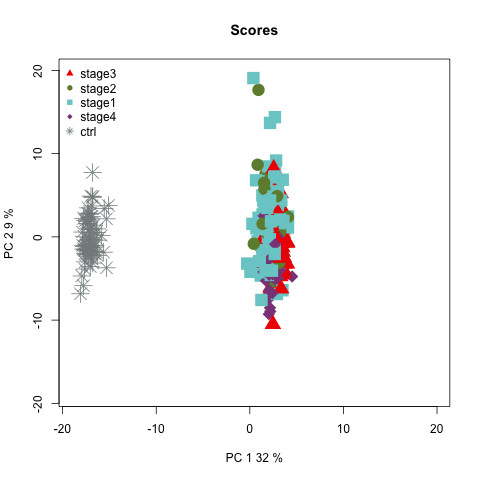

Supplement: Supplementary Material S2 — Quality control and normalization results for gene expression in the five phenotypes. This zip file contains PCA plots before and after normalization for genes and micro-RNAs. All plots were performed after applying filters reported in 2.3. [file DataSheet_2.zip › QC/PCA-mir-AfterNorm.png]

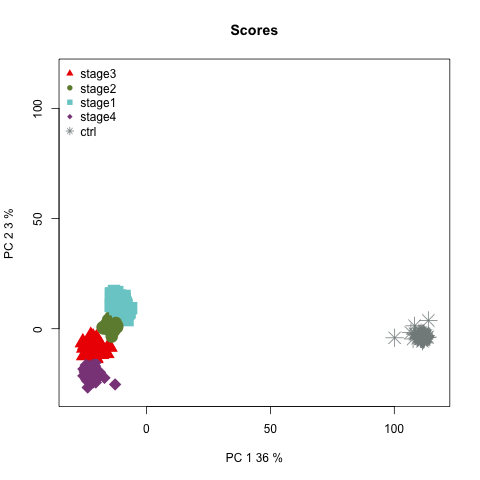

Supplement: Supplementary Material S2 — Quality control and normalization results for gene expression in the five phenotypes. This zip file contains PCA plots before and after normalization for genes and micro-RNAs. All plots were performed after applying filters reported in 2.3. [file DataSheet_2.zip › QC/PCA-rna-AfterNorm.png]

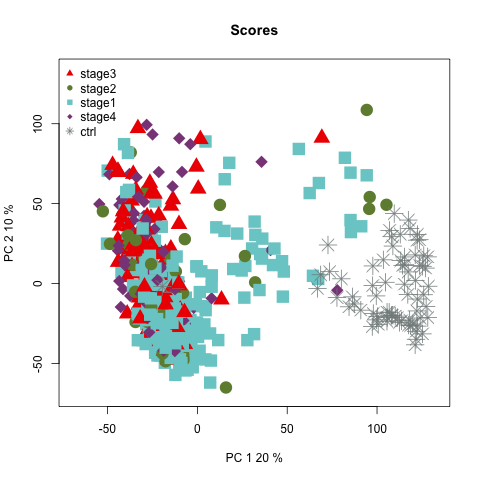

Supplement: Supplementary Material S2 — Quality control and normalization results for gene expression in the five phenotypes. This zip file contains PCA plots before and after normalization for genes and micro-RNAs. All plots were performed after applying filters reported in 2.3. [file DataSheet_2.zip › QC/PCA-rna-BeforeNorm.png]
